# Designing a microbial fermentation-functionalized alginate microsphere for targeted release of 5-ASA using nano dietary fiber carrier for inflammatory bowel disease treatment

**DOI:** 10.1186/s12951-023-02097-6

**Published:** 2023-09-23

**Authors:** Lei Qiu, Renbin Shen, Lei Wei, Shujuan Xu, Wei Xia, Yan Hou, Jinxin Cui, Rong Qu, Jiale Luo, Jian Cao, Jie Yang, Jing Sun, Ronglin Ma, Qiang Yu

**Affiliations:** 1 Department of Gastroenterology, The Affiliated Suzhou Hospital of Nanjing Medical University, Suzhou Municipal Hospital, Gusu School, Nanjing Medical University, Suzhou, 215002 Jiangsu China; 2 Institute of Medical Biotechnology, Suzhou Vocational Health College, Suzhou, 215009 Jiangsu China; 3 Department of Pathology, The Second Affiliated Hospital of Soochow University, Suzhou, 215004 Jiangsu People’s Republic of China

**Keywords:** Nano dietary fibers, Colitis targeting, Gut microbiota, 5-ASA, Gel microspheres

## Abstract

Patients with inflammatory bowel disease (IBD) always suffer from severe abdominal pain and appear to be at high risk for colorectal cancer. Recently, the co-delivery of targeted drugs and gut microbiota has developed into an attractive strategy. A new strategy using gut microbiota fermentation to overcome the interspace diffuse resistance from the mucus layer to control drug release in inflammatory bowel sites (IBS sites) has not yet been available. Here, we designed an alginate hydrogel microsphere encapsulating bifidobacterium (Bac) and drug-modified nanoscale dietary fibers (NDFs). The hydrogel microsphere is responsible for protecting drugs from acidic and multi-enzymatic environments and delivering drugs to the colorectum. Subsequently, the fermentation of Bac by digesting NDFs and proteins as carbon and nitrogen sources can promote drug release and play a probiotic role in the gut microbiota. In vitro evidence indicated that small-sized NDF (NDF-1) could significantly promote short-chain fatty acid (SCFA) expression. Notably, NDF-1 hydrogel microspheres showed a boost release of 5-ASA in the IBS sites, resulting in the amelioration of gut inflammation and remodeling of gut microbiota in chronic colitis mice. This study developed a controlled release system based on microbial fermentation for the treatment of IBD.

**Supplementary Information:**

The online version contains supplementary material available at 10.1186/s12951-023-02097-6.

## Introduction

Inflammatory bowel disease (IBD), including ulcerative colitis and Crohn’s disease, is characterized by chronic, relapsing gastrointestinal inflammation and is becoming increasingly prevalent [1–3]. The clinical therapy for IBD is based on oral or intravenous administration of drugs (*i.e.*, aminosalicylates [4], corticosteroids [5], probiotics [6] and immunosuppressants [7]), which is always accompanied by several side effects or off-target toxicity [8, 9]. To achieve targeted delivery to IBS sites [10], some therapeutic approaches are burgeoning [11], including hydrogel systems [12–14], polymeric nanogels [15], metal nanoparticles [10], oxidation-responsive nanoparticles [16], and other nanocomposites [17–19]. Among them, hydrogel systems (alginate [20], chitosan [21, 22] and hyaluronic acid [23]) have been widely used as a platform for targeting the colorectum to overcome the barriers from the gastric *pH* and degrading enzymes [24]. However, these systems are commonly used to passively target the whole colorectum. Targeting delivery systems that can precisely control drug release in IBS sites are rarely reported [25].

The primary metabolism of gut microbiota is able to breakdown indigestible dietary fibers (DFs) into monosaccharides [26, 27]. It was speculated that targetable drug release could be realized by microbial fermentation in IBS sites to overcome interspace diffuse resistance. Notably, Bifidobacterium (Bac) is one of the common drugs used in the clinical treatment of IBD to promote gut microbiota homeostasis [28, 29]. Therefore, to enhance the fermentability of gut microbiota and promote drug release, DFs, as regulators of microbiota fermentation, may be a desirable drug delivery carrier [30, 31]. In addition, DFs are able to conjugate with drugs covalently or noncovalently due to abundant side chains (esterification) in their branch [32]. Moreover, DFs can react with the mucus layer on the epithelial surface due to specific physicochemical properties (solubility, viscosity, and fermentability) [33]. After entering the colorectum, DFs will be decomposed as a carbon source for the gut microbiota [34], which is further conducive to the release of drugs.

As is well known, dietary fibers are fermented by gut microbiota, and thus give rise to the microbial metabolites, including short-chain fatty acids (SCFAs). Various studies have shown that intake of viscous dietary fiber effectively slow progression of inflammatory bowel disease [35]. However, it has been rarely reported that DF was used as a carrier for oral drug delivery to control the release of drugs. More importantly, this kind of strategy which Bac and drug-modified nano-scaled dietary fibers were together encapsulated into an alginate hydrogel to facilitate the coordinated delivery of them after oral administration has never been attempted.

**Scheme 1:**
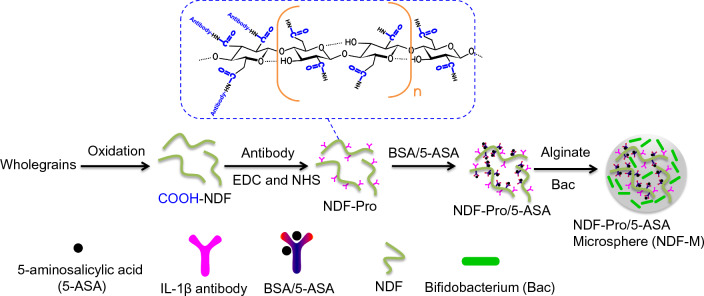
Preparation of an NDF-Pro/5-ASA composite microsphere (NDF-M). First, NDFs were extracted and prepared from wholegrains. Second, IL-1β antibodies and BSA/5-ASA were immobilized sequentially to the surface of NDFs by amide bonds to form NDF-Pro/5-ASA. Finally, both NDF-Pro/5-ASA and Bac were wrapped to form NDF-M in the presence of alginate by an electrostatic droplet generator

**Scheme 2:**
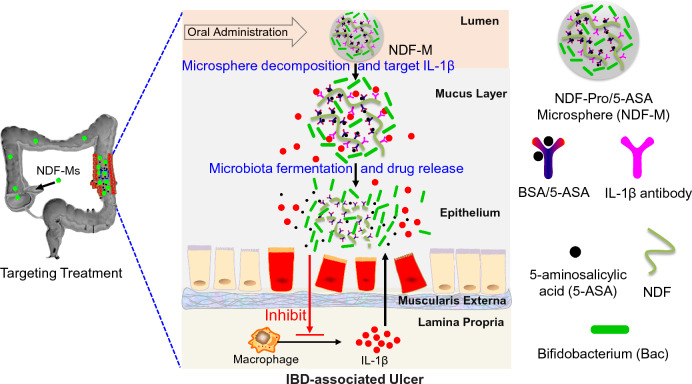
A sketch showing the 5-ASA release process induced by Bac fermentation after NDF-M oral administration. First, gel microspheres are used to overcome the barriers of gastrointestinal digestible enzymes and *pH* and make NDFs and Bac within the microspheres leak into the colorectum after oral administration. Then, NDF-Pro/5-ASA can target the inflamed colon through IL-1β antibodies due to the in situ accumulation of IL-1β inflammatory factors at the site of inflammation. Last, the fermentation of Bac, feeding on NDFs and proteins as carbon and nitrogen sources, respectively, will overcome the interspace diffuse resistance from the mucus layer to promote the release of 5-ASA. Meanwhile, the improvement of gut microbiota would further alleviate inflammation

In our study, we combined the advantages of DFs and gut microbiota to engineer an alginate hydrogel microsphere encapsulating Bac and drug-modified nanoscale dietary fibers (NDFs). NDFs with different length, including NDF-1, NDF-2 and NDF-3, were modified with IL-1β antibodies and BSA/5-ASA (the compound of BSA and 5-ASA via electrostatic interaction) to form NDF-Pro/5-ASA. Subsequently, both NDF-Pro/5-ASA and Bac were wrapped together to form a hydrogel microsphere (NDF-M) in the presence of alginate by an electrostatic droplet generator (Scheme 1). The 5-ASA release from the delivery system may involve four steps: (i) NDF-Pro/5-ASA and Bac are delivered by gel microspheres in the colorectum after oral administration; (ii) NDF-Pro/5-ASA is capable of targeting IBS sites due to IL-1β antibodies upon gel microsphere swelling and decomposition[36]; (iii) 5-ASA is gradually released through the depletion of NDFs and proteins, which is essential for Bac fermentation; (iv) Improving the gut microbiota within IBS areas could potentially mitigate inflammatory response (Scheme 2). Thus, this paper is aimed to uncover the relationship between gut microbiota fermentation and drug release, and is expected to achieve better therapeutic benefits for improving the balance of gut microbiota and alleviating IBD compared to that observed in chronic colitis mice treated with 5-ASA alone. This kind of strategy may serve as a IBD therapy option.

## Methods and materials

### Reagents and materials

We purchased dextran sulphate sodium (DSS, MW: 36,000–50,000) from Thermo Scientific (Waltham, USA) and Hoechst 33,342 from InvivoGen (San Diego, USA). Tumor necrosis factor-α (TNF-α) and interleukin-1b (IL-1β) ELISA Kits were obtained from BD Biosciences (San Jose, USA). Cy5.5 labeled BSA (Cy5.5-BSA) and IL-1β antibodies were purchased from Bioss (Beijing, China). FITC-labeled CD11b antibodies, PE-labeled F4/80 antibodies and PE-labeled Ly6G antibodies were purchased from Biyuntian Biotechnology (Shanghai, China). Bovine serum albumin (BSA), 5-aminosalicylic acid (5-ASA), sodium alginate, N-(3-Dimethylam- inopropyl)-N′-ethylcarbodiimide hydrochloridecommercial grade (EDC) and NHS were from Sigma-Aldrich (St. Louis, USA). MTS kit was from Promega (Madison, USA). Collagenase and DNase were from Roche (Basel, Schweiz). Myeloperoxidase (MPO), catalase (CAT) and malondialdehyde (MDA) assay kits were purchased from Nanjing Jiancheng Bioengineering Institute (Nanjing, China). Other chemical reagents (> 99.5%) were bought from Aladdin Chemical Reagent (Shanghai, China).

### The synthesis and characterization of NDFs

#### The synthesis of NDFs

NDFs including NDF-1, NDF-2 and NDF-3 were extracted from oat flour by chemical and hydrothermal pre-treatments with mechanical defibrillation by high pressure homogenization [37]. In brief, 20 g of oat flour was soaked in ethyl acetate (1:4, w/w) at 25 °C for 3 h, then rinsed with DI H_2_O to remove the ethyl acetate and resuspended with 100 mL with DI H_2_O. The resuspension was immediately adjusted to *pH* 5.5 using 10 M Na_2_CO_3_ solutions. Next, 1.0 g of α-amylase was added and shook for 1 h at 95 °C at 200 rpm. To further remove impurities, the *pH* of the solution was adjusted to 7.0 with 10 M Na_2_CO_3_, and 0.3 g of neutral protease were added and shook for additional 2 h at 55 °C at 200 rpm. Subsequently, the dietary fiber precipitates were collected by centrifugation at 16,000 rpm for 10 min, washed twice times with DI H_2_O and filtered through 70 μm filters, then dried using vacufuge at 60 °C and weighed. Last, three DF powder with the same weight were dissolved in 64% sulfuric acid and shaken for 2 h, 4 h or 8 h at 50 °C and 120 rpm respectively. The resulting DFs were washed with DI H_2_O to neutrality and sonicated by a 1.8 kW power ultrasonic processor (JY99-IIDN, Xinzhi Biotechnology, China) for 2 h with 10 mm head to obtain NDFs with different lengths.

#### The characterization of NDFs

The chemical compositions of NDFs were analyzed by some classical detection methods [37]. Resistant maltodextrin (*AOAC method* 2001.03), β-glucan (*AOAC method* 995.16) and total dietary (*AOAC method* 985.29) were determined according to Association of Official Analytical Chemists procedures (*AOAC*, 2005). Resistant starch was determined by *Chinese NY/T method* 2638.

The morphologies of NDFs were observed by an atomic force microscopy (AFM) (Nanoscope Icon, Veeco, USA). Drops of NDFs solution (50 μg/mL) were placed on a mica plate and dried at room temperature for AFM observations. Zeta potentials and particle lengths of NDFs in water were measured by dynamic light scattering (Zetasizer Nano ZS90, Malvern, UK). Fourier transform infrared (FT-IR) spectroscopy (Tensor 27, Bruker, Germany) was used to confirm the existence of large carboxylic groups on the surface of NDFs.

### Preparation of BSA/5-ASA and surface modification of NDFs

#### Preparation and concentration detection of BSA/5-ASA

A total of 0.25 mL of 4.0 mg/mL BSA and a series of different concentrations (0.25 mL, 0–9.6 mg/mL) of 5-ASA were mixed for 48 h. The mixed solution was passed through an 8000 molecular weight dialysis bag (MD10, Solarbio, China) to remove the free 5-ASA. The contents of BSA and 5-ASA in prepared BSA/5-ASA were detected by a BCA assay kit and fluorescence spectroscopy. Fluorescence spectra was obtained at excitation and emission wavelengths of λex = 260 nm and λem = 300 ~ 600 nm by a microplate reader (Synergy NEO HTS, Biotek, USA).

#### The IL-1β and BSA/5-ASA modifications on the surface of NDFs

IL-1β and BSA/5-ASA were conjugated with NDFs by an amidation reaction [38]. In detail, 100 mg of EDC and 200 mg of NHS were added into 1.0 mL NDFs (1.0 mg/mL in DI H_2_O) and stirred for 2 h at 25 °C. Then NDF pellets were reacted with 1.2 mL of IL-1β solution (10 μg/mL) under magnetic stirring. After 6 h reaction, 1 mL BSA/5-ASA (BSA 37.4 mg/mL, ASA 60.9 mg/mL) was further added into the mixture to synthesize NDF-Pro/5-ASA. NDF-Pro/5-ASA were washed three times with DI H_2_O to remove the free IL-1β and BSA/5-ASA, and suspended in 1.0 mL DI H_2_O. IL-1β and BSA were detected by a BCA kit, and 5-ASA was detected by fluorescence spectra.

### The drug loading efficiency and release efficiency of NDF-Ms

#### The preparation of NDF-Ms and encapsulation efficiency of NDF-Pro/5-ASA

1 mL mixture containing 10 mg sodium alginate, 10 mg Bac and a serial weight of NDF-Pro/5-ASA were mixed and extruded into CaCl_2_ solution (0.1 M) through a 0.5 mm needle with an electrostatic droplet generator (Bio-leader Incorp, China) to form 0.5 mL NDF-M gel microspheres. After gelling for 10 min, the NDF-Ms were collected by natural sedimentation, and immersed in 10 mL 0.05% (w/v) polylysine solution (PLL) in a shaker at 50 rpm for 10 min to form a PLL coating shell on the NDF-M surface. The coated microcapsules were resuspended in 5 mL PBS and counted under a microscope (around 300 microspheres/mL). To examine the drug loading efficiency, 1 mL NDF-Ms microspheres were dissolved with 50 μL 10 M Na_2_CO_3_. The protein concentrations were determined by a BCA kit. Then, the concentrations of 5-ASA in the presence of 1.0 mg/mL proteins were determined by detecting the fluorescence intensity with 260 nm excitation wavelength and 500 nm emission wavelength. The encapsulation efficiency (%) of NDF-Pro/5-ASA was exhibited based on the concentrations of BCA.

#### The release efficiency of NDF-Pro/5-ASA

Simulated gastric and intestinal fluid (SGIF) was used to mimic the in vivo* pH* environments [39]. Briefly, 5.0 mL NDF-Ms were incubated with 12 mL of SGIF to be seeded into 24-well plates (650 μL/well, around 55 microspheres/well), in which there were a total of 18 time points over the 24 h period. At specific incubation time intervals for various wells, the supernatant was extracted by centrifugation (3000 rpm, 5 min), and then 0.5 mL of PBS containing 20 μL of 10 M Na_2_CO_3_ was introduced into each well to dissolve the NDF-Ms. Then the contents of protein, 5-ASA, NDFs and Bac within NDF-Ms were detected by different methods. It was worth noting that we adjusted pH from 1.2 to 5.0 by 10 M Na_2_CO_3_ at 2 h. Similarly, the *pH* was adjusted to 7.0 and 1 mL MRS medium per well was added for the growth of Bac for 6 h incubation.

#### The detection of various ingredients of NDF-Pro/5-ASA

Detection methods for protein and 5-ASA have been previously described. Proteins and 5-ASA were quantified by the BCA kit and fluorescence intensity detection at 500 nm. Bacterial growths were observed by measuring the optical density (OD) at 595 nm. Colony-forming units (CFUs) were determined to visualize the bacterial cells. For the detection of remaining NDFs, the samples collected at each time points and washed three times by centrifugation at 10,000 rpm for 10 min with DI H_2_O to remove alginate and BSA/5-ASA, and suspended in 400 μL DI H_2_O. Then 250 μL sulfuric acid solutions containing 0.025 M borax, as a catalyst of decomposition of NDFs, were added into the solution to get glucuronic acid. Glucuronic acid would react with 0.05 mL 0.1% imidazole to form a complex of which the absorbance at 530 nm was proportional to the glucuronic acid concentration. A standard curve of glucuronic acid (0.025 ~ 0.8 mg/mL) was used to calculate the concentration of uronic acids.

#### Bifidobacterium (Bac) Culture

Bifidobacterium (ATCC 15707) was cultured in MRS broth for 24 h under anaerobic conditions at 37 °C. Bac was purified by centrifugation (10,000 rpm, 5 min, 4 °C) and resuspended with phosphate-buffered saline (PBS, *pH* 7.4), and the concentration of Bac was adjusted to approximately 10 mg/mL.

#### Short-chain fatty acids quantification analysis of fermentation broth

Short-chain fatty acids, including acetate, propionate, lactate, butyrate, valerate and isovalerate were measured by LC–MS/MS spectrometric detector [40]. The derivatizing reagents were 12 mM EDC, 15 mM 3-Nitrophenylhydrazine and pyridine (2% v/v) in methanol. The reaction was stopped with a quenching reagent consisting of 0.5 mM beta-mercaptoethanol and 0.1% formic acid in water. Colon contents dissolved with saline according to 1.0 mL per 10 mg, and 5 μL was mixed with derivatizing reagent (100 μL) and incubated for 1 h at 4 °C. Then, the samples were centrifuged at 16,000 rpm for 10 min at 4 °C, and 20 μL of supernatant was mixed with 200 μL of the quenching reagent. After centrifugation at 16,000*g* for 10 min at 4 °C, supernatants were collected for LC–MS/MS analysis. A quadrupole-time of flight mass spectrometer (Q-TOF, Agilent, Suzhou, China) operating in negative ion mode was coupled to C18 chromatography via electrospray ionization and used to scan from m/z 100 to 300 at 1 Hz and 15,000 resolution. LC separation was equipped with an Acquity UPLC BEH C18 column (2.1 mm × 100 mm, 1.75 um particle size, 130 A° pore sizes; Waters, Milford, MA) using a gradient of solvent A (0.01% formic acid in water) and solvent B (0.01% formic acid in isopropanol). Autosampler temperature was 5 °C, and the injection volume was 10 μL. Ion masses for derivatized acetate, propionate, lactate, butyrate, valerate and isovalerate were 194, 208, 224, 222, 236 and 236, respectively.

### Cell culture and ELISA and cytotoxicity test

#### Cell culture

SW 480 cells and RAW 264.7 cells were purchased from the Institute of Biochemistry and Cell Biology (Shanghai, China). Cells were cultured in DMEM medium supplemented with 10% FBS, 100 U/mL of penicillin and 100 μg/mL of streptomycin at 5% CO_2_, 37 °C.

#### ELISA and cytotoxicity test in RAW264.7 cells exposed to the fermentation broth

RAW264.7 cells were incubated with different volumes of fermentation broth for 24 h. the fermentation broth was obtained for further research when NDF-Ms were exposed to SGIF according to 2.4.2 in this section for 24 h. The supernatants were collected to detect the expression of cytokines using ELISA kits. Meanwhile, aliquots of 120 μL diluted MTS solutions were added to 96-well plates containing cell pellets and incubated at 37 °C for 3 h. Then the supernatants (100 μL/well) were transferred into new plates for absorbance detection at OD 490 nm by a Microplate Reader (Synergy NEO HTS, Biotek, USA).

### Induction of chronic colitis and drug treatments in mice

#### Induction of chronic colitis mice and the grouping of mice

Eight week-old male C57BL/6 mice, obtained from Jiangsu GemPharmatech Biological Technology (Nanjing, Jiangsu, China), were housed in a standard laboratory conditions by Nanjing Medical University guidelines for care and treatment of laboratory animals. To induce chronic colitis in mice, C57BL/6 mice were administered with 1.5% (w/v) DSS in their drinking water and libitum for three consecutive 5-day periods: days 1–5, 11–15, and 21–25. After each period of DSS administration, the mice with chronic colitis were divided into six groups (6 mice/group) and treated with saline, 5-ASA (2.0 mg/20 g), Bac (10 mg/20 g), NDF-M1/L, NDF-M1/M or NDF-M1/H by oral gavage. NDF-M1/L contained 1.0 mg NDFs, 0.61 mg proteins, 1.0 mg 5-ASA and 4.0 mg Bac. NDF-M1/M and NDF-M1/H were increased by 2- and fourfold compared to NDF-M1/L. Besides, the healthy mice as the control group were allowed to drink water. On day 31, all mice were sacrificed under isoflurane anesthesia.

#### Routine evaluation indexes of NDF-M1s for the treatment of colitis

Fecal bleeding, changes in body weight, and visible stool consistency were assessed every 3 days. Disease activity index (DAI) was the summation of the stool consistency index (0–3), fecal bleeding index (0–3), and weight loss index (0–4). Besides, the entire colon (from the cecum to the rectum) was excised and the length of the colon was measured, opened longitudinally and colon contents were collected and used for further investigation. Blood samples were collected for the quantification of hematological parameters and biochemical markers relevant to liver/kidney functions. For serum biochemical assessment, blood samples were collected from the orbital venous plexus, and the plasma was obtained by centrifugation at 1000 rpm for 10 min at 37 °C. The serum biochemical parameters, including alanine aminotransferase (ALT), aspartate aminotransferase (AST), blood urea nitrogen (BUN) and creatinine (CREA), were measured using an automatic chemistry analyzer (AU480; Beckman Coulter, USA).

#### The expression levels of inflammatory-related molecular

The colonic tissues were collected and homogenized in cold saline immediately after the mice euthanization. A portion of the colon tissue homogenate was taken for measuring the levels of TNF-α and IL-1β by ELISA kits, and the levels of MDA, CAT and MPO activity according to their manufacturer's instructions. Briefly, stored tissues were homogenized in phosphate buffer (*pH* 7.4) at a ratio of 1:10 (per mg in 10 mL). The supernatant was collected by centrifugation at 12,000 rpm for 15 min at 4 °C. In addition, the total protein concentration was measured by the BCA method. Besides, immunohistochemistry analysis was also conducted to examine the expression of IL-1β in the colon. The colon sections in paraffin were deparaffinized and immersed in 0.3% H_2_O_2_-PBS buffer for 1 h to eliminate the interference of endogenous peroxidase. After washing three times, the sections were blocked in 10% BSA containing 0.25% Triton X-100. After secondary antibody incubation and substrate reaction, the final immunohistochemistry images were captured by a Nikon microscope (Nikon Eclipse Ni, Nikon, Japan).

#### Histological assessment

Initially, a 1 cm segment of the distal colon was preserved by fixation in 4% (v/v) buffered formalin and then embedded in paraffin. Tissue sections with a thickness of 7 μm were stained with hematoxylin and eosin (H&E). The histology of the colon was visualized by optical microscopy. Besides, major organs including heart, liver, spleen, lung, kidney, stomach and intestine, were harvested and fixed in 4% (v/v) buffered formalin and embedded in paraffin to assess the acute toxicity of NDF-M1/H.

#### Confocal imaging observation and flow cytometer detection for neutrophils and macrophages related to inflammation remission

To prepare single-cell suspensions, colons isolated from mice were cut into pieces with the length at 0.5 cm after the fat was dissected away. Cleaned colon fragments were incubated with 5 mM EDTA, 1 mg/mL collagenase and 25 μg/mL DNase at 250 rpm for 20 min at 37 °C. Then cell suspensions were passed through a 70 μm cell strainer. One part of the single-cell suspensions was stained with Hoechst 33,342 (blue), FITC-labeled CD11b antibodies (green) and PE-labeled Ly6G antibodies (red) for 2 h to visualize the nuclei and neutrophils by CLSM (Leica, Germany). The other was stained with Hoechst 33,342 (blue), FITC-labeled CD11b antibodies (green) and PE-labeled F4/80 antibodies (red) to visualize the nuclei and macrophages, respectively. Flow cytometric analysis was performed using a BD FACSVerse flow cytometer, and data were analyzed using FlowJo 7.6 software.

#### DNA extraction and 16S rRNA gene sequence analysis of colon contents

The colon contents were dissolved with the normal saline according to 1 mL per 10 mg colon contents. All the sequencing procedures including quality inspection of samples, DNA extraction, 16S rRNA tag-encoded high-throughput sequencing and general data analyses were performed by Azenta Life Sciences (Suzhou, China) [24]. In brief, total bacterial DNA was extracted by Qubit® dsDNA HS Assay Kit as the manufacturer’s instructions. Upstream primer 5′-CCTACGGRRBGCASCAGKVRVGAAT-3′ and downstream primer 5′-GGACTACNVGGGTWTCTAATCC-3′ were used to amplify the V3–V4 regions of 16S rRNA gene. The concentration was detected by a microplate reader (Tecan, Infinite 200 Pro) and the fragment size was detected by 1.5% agarose gel electrophoresis which was expected at ~ 600 bp. Sets of sequences with 97% similarity were assigned as an OTU using VSEARCH software, and a representative sequence for each OUT was employed to annotate the taxonomic information in Mothur with the RDP classifier (Ribosomal Database Program). Based on OTU analysis, Chao1 alpha diversity index calculated by QIIME software reflect the species richness and evenness. NMDS based on the distance between the matrix Brary–Curtis displays beta diversity visualization.

### Induction of acute colitis and pharmacokinetic analysis of NDF-M1/H

Eighteen mice including nine control mice and nine acute colitis mice (induced by 3% DSS for 7 days) were divided into six groups (3 mice/group). They were treated with saline, 4.0 mg 5-ASA, or NDF-M1/H for 12 h, respectively. Subsequently, all mice were sacrificed under isoflurane anesthesia to collect serum, colon contents and colon tissues. To observe the pharmacokinetics of NDF-M1/H, the dissociated colon tissues were first classified as IBS sites and non-inflammatory bowel sites (non-IBS sites). The expression of IL-1β was detected by the ELISA kit. Then their tissue homogenates and colon contents diluted with saline (1:10) were further conducted to the determination of 5-ASA, proteins and glucuronic acids (GLCUA, the decomposition of polysaccharides) by previous described methods.

### In vivo and in vitro bioluminescence imaging

The BSA was replaced with Cy5.5-BSA during the NDF-Pro/5-ASA synthesis, which were wrapped together to form Cy-5.5 labeled NDF-M1/H (Cy5.5-NDF-M1/H) gel microspheres. Normal mice and acute colitis mice were orally administered with Cy5.5-NDF-M1/H at the same dose. After 24 h, Cy5.5 fluorescence images from the mice as well as the isolated colorectal tissues were detected by IVIS^®^ Spectrum (IVIS Spectrum, PerkinElmer, USA) and analyzed using Living Image software. Subsequently, the colorectal tissues with Cy5.5 fluorescence were fixed in 4% (wt/vol) paraformaldehyde at 4 °C overnight for the H&E and immunohistochemistry staining.

### Statistical analysis

The R values of correlation coefficients between the curve of Bac growths and the curve of 5-ASA release curve, protein decomposition and GLCUA residual were calculated by one-way ANOVA analysis. The heatmap was completed by the software “Heatmap Illustrator”. All of the results were shown by mean ± SD. Statistical significance was evaluated using one-way ANOVA or two-tailed Student’s *t* test.

## Results

### Preparation and characterization of NDF-Ms

**Fig. 1:**
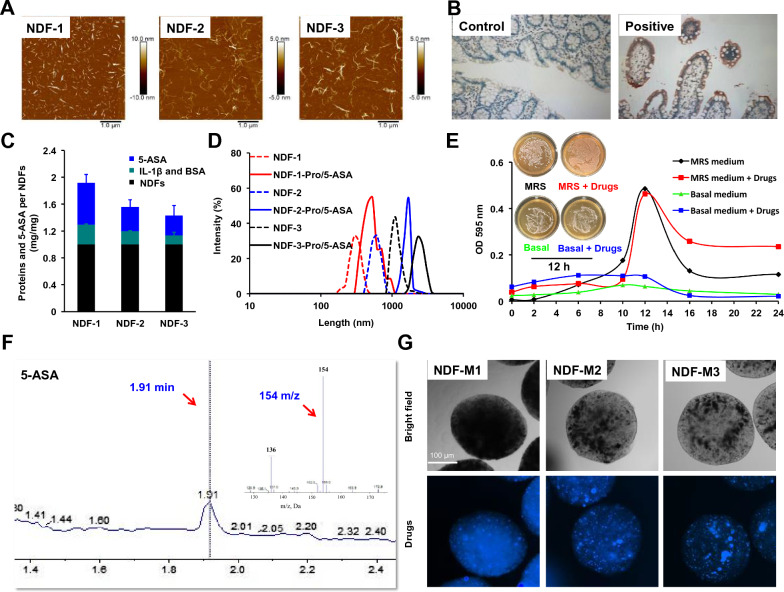
Preparation and Characterization of NDF-Ms. **A** Visualizing the morphologies of NDFs by AFM images. A drop of NDF suspension (50 μg/mL) was placed on a mica plate and then dried at room temperature for AFM observation. **B** Immune staining of IL-1β distributed in the colorectum in wild-type mice and mice with gross enteritis. **C** Binding efficiency of proteins and 5-ASA on the surface of NDFs (mg/mg). Proteins and 5-ASA were quantified by a BCA Protein Assay kit (BCA kit) and fluorescence intensity detection at 500 nm. Data are displayed as the mean ± SD (n = 3 independent samples). **D** Hydrodynamic length changes from NDFs to NDF-pro/5-ASA in DI H_2_O solution. The changes demonstrated the synthesis of intermediate NDF-pro/5-ASA. **E** Bacterial growths exposed to NDF-Pro/5-ASA were observed in the MRS medium or basal medium by measuring the optical density (OD) at 595 nm. CFUs were determined at 12 h to visualize the bacterial cells. **F** Qualitative analysis of 5-ASA. 5-ASA was detected and qualitatively analyzed at 1.91 min (m/z = 154 for 5-ASA, red arrow) by LC − MS/MS. **G** Representative images of NDF-Ms. NDF-M microspheres were prepared by extruding 1.5% w/v alginate solutions containing NDF-Pro/5-ASA and Bac into CaCl_2_ solution (0.1 M) with an electrostatic droplet generator. NDF-M microspheres were captured to visualize NDF-Pro/5-ASA distributions by confocal microscopy

NDFs were extracted and prepared from wholegrains according to a modified synthetic procedure [41]. AFM was performed to characterize their sizes and morphologies, which suggested NDFs with lengths of 100–500 nm, 500–1000 nm, and 1000–2000 nm, denoted NDF-1, NDF-2, and NDF-3, respectively (Fig. 1A). To make the chemical characterization of natural polysaccharides clearer, we determine the chemical composition of NDFs. The results were as shown in Table 1. Firstly, above 80% NDFs were present in the whole oat flour extract. Of these, β-glucan, resistant maltodextrin and resistant starch accounted for 70–75%, 10–15% and 3–4%, respectively. Furthermore, it is also known that the surface of these polysaccharides possesses a great deal of functional hydroxyl groups and is modified more frequently after functioned with EDC and NHS coupling reagents [42], so that NDFs become more accessible to the drug. FT-IR spectra of COOH-NDF and NDF-Pro/5-ASA in Additional file 1: Figs. S1A and S1B showed the characteristic absorption peaks at ~ 1,600 cm^–1^ reflecting the stretching vibration of the carbonyl group (–C = O) and at ~ 1,560 cm^–1^ reflecting the –NH bending vibrations [41]. It showed the binding of IL-1β antibodies and BSA/5-ASA on the surface of NDFs by an amide bond. Among them, IL-1β antibody modification was expected to target IL-1β inflammatory cytokines accumulated at IBS sites of IBD mice, which was confirmed by immunohistochemistry staining (Fig. 1B). BSA was used to bind 5-ASA via electrostatic interactions, and the BSA/5-ASA conjugation data showed that there were 37.4 mg BSA and 60.9 mg 5-ASA per 1 mL DI H_2_O.

**Table 1:** The chemical compositions of NDFs

Compound (g/100 g)	NDF-1 NDF-2 NDF-3		
β-glucan	70.9 ± 1.8	71.5 ± 6.8	74.4 ± 0.8
Resistant maltodextrin	10.7 ± 0.6	11.7 ± 0.6	13.6 ± 1.5
Resistant starch	3.8 ± 0.2	3.5 ± 0.4	3.4 ± 0.1
Total dietary fibers	80.2 ± 0.7	82.0 ± 6.5	86.9 ± 3.8

After the synthesis of BSA/5-ASA, the preparation of NDF-Pro/5-ASA was performed as follows. First, the protein content was examined by the BCA assay kit, which revealed that approximately 10 μg of IL-1β antibodies were combined with 1.0 mg of NDFs (Additional file 1: Fig. S1C). In addition, the functionalization of BSA on NDFs was concentration-dependent (Additional file 1: Fig. S1D). NDF-1 showed the highest BSA binding (0.37 ± 0.02 mg/mg) compared with the other two NDFs (0.23 ± 0.02 and 0.17 ± 0.04 mg/mg). Then, the loading capacity of 5-ASA was determined by fluorescence spectroscopy [43]. As shown in Additional file 1: Fig. S1E, the fluorescence intensity was obviously shifted with different concentrations of 5-ASA. As shown in Additional file 1: Fig. S1F, the loading of 5-ASA increased gradually and reached a plateau with increasing 5-ASA concentration. Based on this curve, the 5-ASA loading concentration in NDF-1 was 0.62 ± 0.12 μg/mg, which was significantly higher than that in the other two NDFs (0.36 ± 0.10 and 0.30 ± 0.15 μg/mg) (Fig. 1C). Additionally, the length and zeta potential of NDF-Pro/5-ASA were increased (Fig. 1D and Additional file 1: Table S1), which suggested the successful synthesis of NDF-Pro/5-ASA. Herein, we obtained three different species of NDF-Pro/5-ASA, denoted NDF-1-Pro/5-ASA (1.0 mg/0.37 mg/0.62 mg), NDF-2-Pro/5-ASA (1.0 mg/0.23 mg/0.36 mg) and NDF-3-Pro/5-ASA (1.0 mg/0.17 mg/0.30 mg).

After that, NDF-Pro/5-ASA and Bac were coencapsulated by alginate-based gel microspheres, which were expected to protect 5-ASA and Bac in the detrimental gastrointestinal environment. Then, the Bac fermentation activity was examined by colorimetry and CFU assays. As shown in Fig. 1E, the growth of Bac in the presence of high NDF-Pro/5-ASA concentrations was similar to the control group in MRS medium. Interestingly, the proliferation curve of Bac in the presence of NDF-Pro/5-ASA was slightly faster than that of the control group at the initial stage of culture in basal medium without carbon and nitrogen sources (Fig. 1E), which suggested that NDF-Pro/5-ASA may partially provide carbon and nitrogen sources. Next, the structure of 5-ASA was identified by LC‒MS/MS (Fig. 1F), which showed that the molecular structure of 5-ASA was not changed. It can be concluded that Bac fermentation can be supplied by NDFs and proteins as carbon and nitrogen sources but has no influence on the 5-ASA structure. To overcome the barriers of gastrointestinal enzymatic and acidic environments, three different types of NDF-Pro/5-ASA and Bac were individually encapsulated into alginate hydrogel microspheres named NDF-Ms (NDF-M1, NDF-M2 and NDF-M3) by an electrostatic droplet generator [44]. To optimize the encapsulation capacity of NDF-Pro/5-ASA and Bac, we mixed NDF-Pro/5-ASA at different concentrations of 1, 2, 5, 10, and 20 mg/mL (based on 5-ASA), 10 mg/mL Bac and 10 mg/mL alginate. The mixture was individually wrapped into alginate hydrogel microspheres and washed with PBS, and the concentrations of 5-ASA within the microspheres were determined by fluorescence spectroscopy. Table 2 data showed that all 5-ASA encapsulation efficiencies were maintained at ~ 80%. Based on the administration concentration conversion between the clinical trial and mouse experimentation [45], a ratio of 0.5:1 (5-ASA: alginate) was selected and used to conduct subsequent experimental studies. To further visualize the morphology of the gel microspheres, fluorescence and light microscopy (Fig. 1G) were conducted to show that the sizes of all gel microspheres were approximately 200–300 μm and that the drugs were uniformly dispersed inside the gel microspheres. Therefore, we successfully constructed delivery microspheres encapsulated with Bac and NDF-Pro/5-ASA.

**Table 2:** Encapsulation efficiency (%) for NDFs under the different ratios

NDF-Pro/5-ASA: Alg (mg: mg)	Encapsulation efficiency (%)		
	NDF-M1 NDF-M2 NDF-M3		
0.1: 1.0	84.3 ± 2.0	86.3 ± 6.7	84.1 ± 6.9
0.2: 1.0	85.7 ± 6.3	93.2 ± 4.4	93.1 ± 4.3
0.5: 1.0	90.2 ± 3.6	90.3 ± 0.8	88.0 ± 5.3
1.0: 1.0	90.9 ± 5.8	89.6 ± 3.9	85.3 ± 5.6
2.0: 1.0	81.9 ± 2.7	84.4 ± 2.6	80.5 ± 5.1

### Drug release and Bac fermentation of NDF-Ms in vitro

**Fig. 2:**
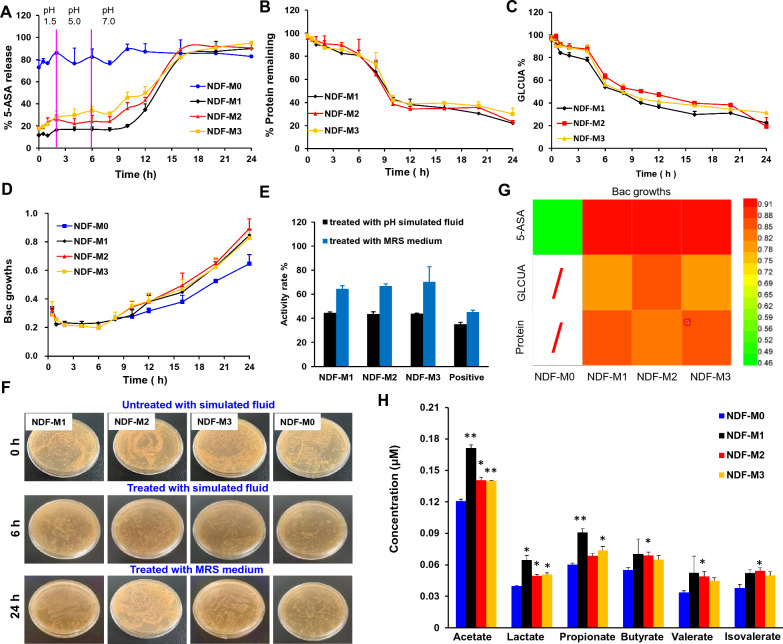
Drug controlled release and Bac fermentation of NDF-Ms in vitro*.* **A** Percentage of 5-ASA release over time. **B** and **C** Percentages of proteins and GLCUA left in NDF-M microspheres over time. The in vitro drug release experiment was conducted using SGIF that was used to mimic the in vivo* pH* environments and transit times of NDF-Ms. 5-ASA, proteins and GLCUA were measured by fluorescence intensity detection, BCA kit and *UV*–vis at 530 nm, respectively. **D** Bac growth was observed after NDF-Ms were exposed to SGIF for 0–24 h by measuring the optical density (OD) at 595 nm. **E** Percentage of Bac at 6 h and 24 h. Data are presented as percentages relative to 0 h. **F** Representative images of Bac colonies at 0 h, 6 h and 24 h. CFUs were further determined at 0 h, 6 h and 24 h to visualize the bacterial cells. **G** Correlation analysis between the curve of Bac growth and the curve of the 5-ASA release curve, protein decomposition and GLCUA residual. The coefficients were displayed by the heatmap. **H** Quantification of the SCFAs. The SCFAs from NDF-M microspheres exposed to SGIF at 24 h were derivatized and measured by LC−MS/MS. These results are reported as the mean ± SD, n = 3. *p < 0.05, **p < 0.01, and ***p < 0.001 versus the NDF-M0 group

The 5-ASA release profile was examined in the presence of SGIF [46]. As shown in Fig. 2A, while the release of 5-ASA was > 70% in the microspheres without NDF combination (NDF-M0) for 24 h, 5-ASA in the three NDF-Ms showed only 30% release during the first 6 h of exposure at *pH* 1.5–5.0 and then had an increased release during 6–16 h at pH 7.0 (Fig. 2A), which suggested that > 70% of the 5-ASA in NDF-Ms could be released at the colorectal section. Meanwhile, the residual proteins and GLCUA in microspheres were examined. As expected, the protein and GLCUA contents were correspondingly reduced (Fig. 2B and C). Therefore, it was strongly evidenced that the targeted delivery of 5-ASA in the colorectum could be realized by the constructed NDF-Ms.

It was speculated that the reduction in proteins and NDFs might be derived from Bac fermentation. To explore this hypothesis, we detected bacterial growth by absorbance measurements at 595 nm and bacterial proliferation by CFU assay. As shown in Fig. 2D, the Bac in the three types of NDF-Ms presented a certain level of reduction for 6 h and then a significant rise for 6 ~ 24 h after exposure to SGIF. In addition, the quantitative results in Fig. 2E show the percent change in the number of Bac at 6 h and 24 h. This result indicated that microspheres showed significant protective effects at 6 h and were favorable for the proliferation of Bac at 24 h. As expected, the CFU assay also revealed the high activity and proliferation of Bac (Fig. 2F).

To confirm the relationship between 5-ASA release, NDF decomposition or proteolytic efficiency, and Bac proliferation, we calculated the correlation coefficients (R values) (Additional file 1: Table S2) and integrated the R values in a heatmap (Fig. 2G). Red indicates high correlations between in vitro 5-ASA release, NDF decomposition or proteolytic efficiency and Bac proliferation, while green represents poor correlations. In NDF-M1, the 5-ASA release, NDF decomposition and proteolytic efficiency showed the highest correlations with Bac proliferation after 24 h exposure (Fig. 2G). These results suggested that Bac fermentation facilitated the release of 5-ASA by feeding on NDFs and proteins as carbon and nitrogen sources, respectively.

With the consumption of proteins and NDFs, various metabolites were produced during Bac fermentation. Among them, the most abundant metabolic molecules were short-chain fatty acids (SCFAs). LC‒MS/MS was performed to quantify SCFAs, including acetate, lactate, propionate, butyrate, valerate and isovalerate (Additional file 1: Fig. S2). Further quantitative results in Fig. 2H showed that the contents of all SCFA levels detected in NDF-M1, NDF-M2 and NDF-M3 were higher than those in NDF-M0. Acetate, lactate and propionate in NDF-M1 showed increased expression levels than NDF-M2 and NDF-M3. In addition, other SCFAs, such as butyrate, valerate and isovalerate, did not exhibit obvious changes among NDF-M1, NDF-M2 and NDF-M3. These results regarding the proliferation of Bac and the expression levels of SCFAs revealed that NDF-M1, NDF-M2 and NDF-M3 could enhance the fermentation level of Bac, and NDF-M1 showed a better effect.

### Therapeutic effects of NDF-Ms on chronic colitis

Before evaluating the therapeutic effect of NDF-Ms, we examined cytotoxicity and inflammatory cytokines induced by the fermentation broth of NDF-Ms. Additional file 1: Fig. S3A shows that all fermentation broths at different concentrations had limited effects on the proliferation of RAW 264.7 cells or SW480 cells, suggesting that they have good biocompatibility. Then, we examined the expression of inflammatory cytokines (IL-1β and TNF-α) in RAW 264.7 cells after exposure to fermentation broths. Al(OH)_3_ nanoparticles were used as a positive control to promote the release of inflammatory cytokines [47]. As shown in Additional file 1: Fig. S3B, NDF-M1 induced negligible IL-1β production, similar to the blank control and lower than other NDFs. Based on the previous experimental results and the concentration range of 5-ASA and Bac used in the clinic [45], we selected NDF-M1 and administered three different doses of NDF-M1 at low, medium and high levels (named NDF-M1s, including NDF-M1/L, NDF-M1/M, NDF-M1/H) for the next study. Thus, we further examined the animal acute toxicity of NDF-M1/H at the highest concentration. Additional file 1: Fig. S3C showed little effect on the inflammation of different tissues (*e.g.*, heart, liver, spleen, lung and kidney), suggesting that they have good biocompatibility.

**Fig. 3:**
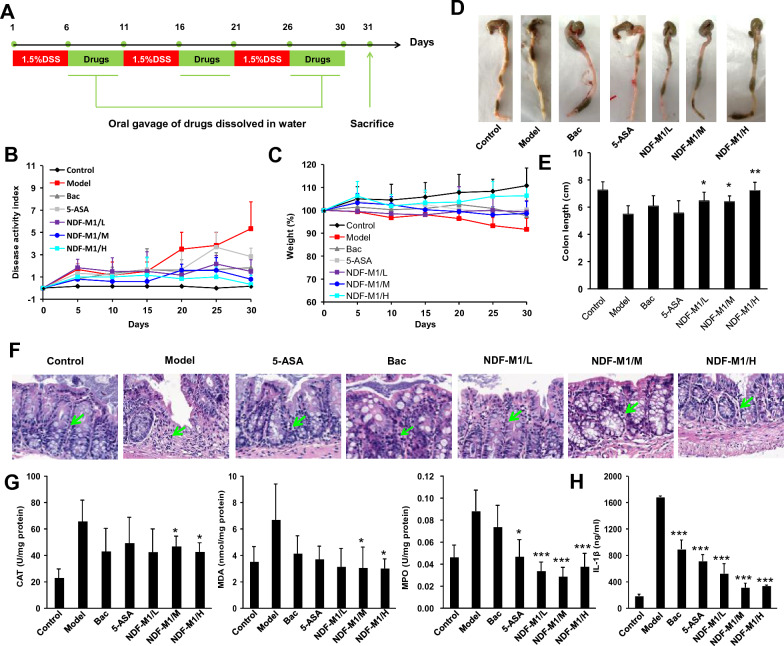
Therapeutic effects of NDF-M1 in mice. **A** Scheme of the treatment procedures. **B** and **C** Changes in body weight and DAI during 30 days of treatment. The scores were graded according to DAI, including rectal bleeding, loose stools and body weight. **D** and **E** Representative photos of colons and colon length statistics. The colon length was measured using Image J. **F** Representative H&E-stained histological sections of colonic tissues. The degree of inflammatory cell infiltration is delineated by green arrows. **G** The levels of oxidative mediators, including MDA, CAT, and MPO. Their production in the colonic tissue homogenates was detected by their respective assay kits. **H** The expression levels of representative IL-1β inflammatory cytokines in colonic tissue, which were detected by ELISA. *p < 0.05 and ***p < 0.001 versus the model group. All data are presented as the mean ± SD (n = 6)

Then, animal experiments, which complied with the guidelines of the Nanjing Medical University’s Regulations of Animal Experiments and were approved by the Animal Experiment Committee of Nanjing Medical University, were conducted to exploit the therapeutic efficacy of NDF-M1s on chronic colitis mice, which was induced by repeated treatment with 1.5% DSS (Fig. 3A). 5-ASA and Bac were used as the control groups. The body weight, DAI and optical images of the colorectum were examined. As shown in Fig. 3B and C, NDF-M1 treatment significantly improved the weight loss of mice and DAI compared to the model group. In addition, the colorectal lengths in the NDF-M1-treated groups were obviously longer than those in the model group, and there were more and heavier contents in the colorectum of the three NDF-M1 groups, suggesting the improvement of dietary behaviors compared to the model group (Additional file 1: Fig. 3D and E). Second, the hematological parameters and biochemical markers were examined to show that total proteins and PLT cells (platelet cells) were obviously increased, while ALT/AST (glutamic pyruvic transaminase to glutamic oxaloacetic transaminase) and Neu cells (neutrophil cells) were obviously decreased after 31 days of treatment with NDF-M1s compared to the model group (Additional file 1: Fig. S4). In addition, as a control of therapeutic efficacy with 5-ASA or Bac, both displayed considerably lower efficacy than NDF-M1s. Interestingly, NDF-M1/M and NDF-M1/H exhibited better efficacy than NDF-M1/L. Subsequently, the inflammation levels were evaluated by histopathological examination of the colonic tissue and the expression of inflammatory cytokines (IL-1β and TNF-α) and inflammatory-related molecular mediators (MDA, H_2_O_2_, and MPO). As shown in Fig. 3F, infiltrations of inflammatory cells (green arrow) in the NDF-M1 treatment group were significantly reduced and had less colonic tissue damage compared to the model group shown by H&E staining. In addition, the expression levels of inflammation-related substances such as MDA, H_2_O_2_, MPO and IL-1β were also decreased (Fig. 3G and H).

**Fig. 4:**
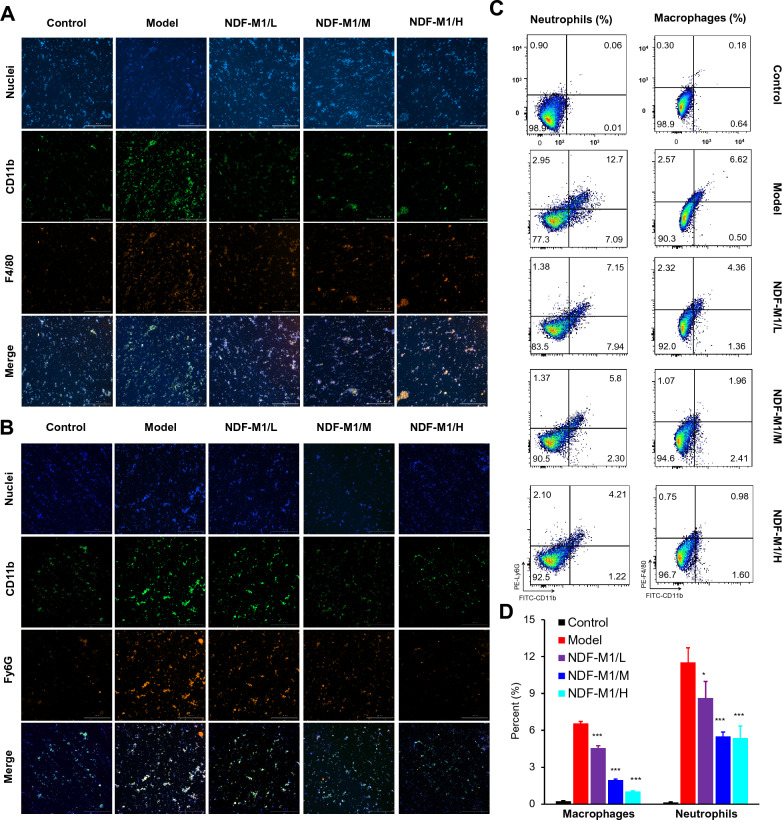
Inflammatory activity analysis of colonic tissues. **A** and **B** Representative confocal images of neutrophils (CD11b^+^ Ly6G^+^) and macrophages (CD11b^+^ F4/80^+^) among all treatment groups. **C** and **D** Flow cytometry dot plots and quantitative analysis of both cell populations. Single-cell suspensions were derived from colonic tissues of mice with colitis after 30 days of different drug treatments. Each single-cell suspension from different treatment groups was divided into two equal parts. One was stained with Hoechst 33,342 (blue), FITC-labeled CD11b antibodies (green) and PE-labeled Ly6G antibodies (red) to visualize the nuclei and neutrophils, and the other was stained with Hoechst 33,342 (blue), FITC-labeled CD11b antibodies (green) and PE-labeled F4/80 antibodies (red) to visualize the nuclei and macrophages. The merge of the three colors represents the number of cells. The number of both cell populations was further quantified by flow cytometric analysis. *p < 0.05 and ***p < 0.001 versus the model group. All data are presented as the mean ± SD (n = 6)

In addition, we tested the recruitment of neutrophils and macrophages related to inflammation remission. The fluorescence intensity of CD11b^+^Ly6G^+^ neutrophils and CD11b^+^F4/80^+^ macrophages in single-cell suspensions of colonic tissues was significantly decreased after 31 days of treatment with NDF-M1s compared to the model group (Fig. 4A and B). To quantify the percentages of the two kinds of cells, we performed a fluorescence quantification study by flow cytometry (Fig. 4C). The quantitative statistical results showed that the fluorescence intensities of CD11b^+^Ly6G^+^ neutrophils and CD11b^+^F4/80^+^ macrophages from NDF-M1/M and NDF-M1/H group mice were significantly decreased compared to those of the model group (Fig. 4D). These data demonstrated that the inflammatory activity of colonic tissues was significantly lower after NDF-M1 treatment.

**Fig. 5:**
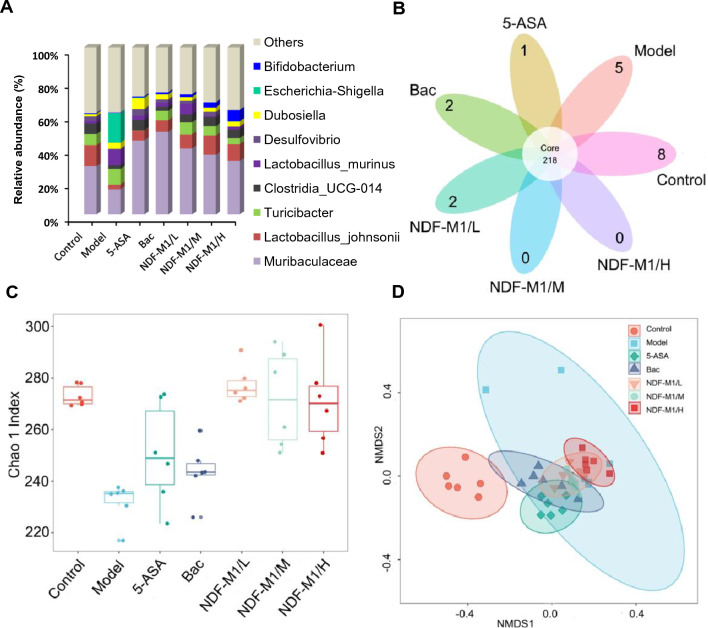
Analysis of the gut microbiota in chronic colitis mice after NDF-M1 treatment. **A** Changes at the genus level in fecal microbiota composition. **B** The observation of operational taxonomic unit (OTU) richness. **C** Box-and-whisker plot of Chao 1 alpha diversity of the microbial community in the feces based on 16S profiling. Boxes show median, first, and third quantiles, and whiskers denote the minimum to maximum range. **D** Nonmetric multidimensional scaling (NMDS) analysis of the microbiota composition. All data are presented as the mean ± SD (n = 6)

To further explore the structural changes in the gut microbiota, we investigated the composition of the gut microbiota by 16S rRNA gene sequencing. Compared to the model group, the chronic colitis mice with NDF-M1 treatment showed a significant decrease in the relative abundance of Lactobacillus_murinus, Escherichia-shigella, Ruminococcus_torques, Blautia, and Clostridium_perfringens and a significant increase in Muribaculaceae, Lachnospiraceae, and Lactobacillus_johnsonii. Importantly, Bifidobacterium, as one of the drug components, had a significant increase, suggesting that it survived well after arriving at the colon (Fig. 5A). Briefly, NDF-M1 treatment promoted the diversity, richness and resilience of the gut microbiota in chronic colitis mice, which was closer to that of the control group mice. In addition, other groups showed a significant loss in bacterial richness, referred to as operational taxonomic unit (OTU) richness, and an unexpected addition that did not exist in normal mice (Fig. 5B). The α-diversity indices of the Chao 1 index evaluating gut microbial community richness and community diversity showed a significant decrease in the model group, whereas treatment with NDF-M1 effectively restored the α-diversity of the gut microbiota (Fig. 5C). Finally, to disclose the degree of similarity of gut microbial composition among all groups, nonmetric multidimensional scaling (NMDS) analysis in the unweighted UniFrac distance showed that the bacterial community structures of NDF-M1 treatment were similar to those of the control mice but distinct from those of the model mice (Fig. 5D). These results suggested that NDF-M1 treatment could remodel the gut microbiota by shifting the microbial community profile from a dysbiotic state toward homeostasis in mice with DSS-induced colitis.

**Fig. 6:**
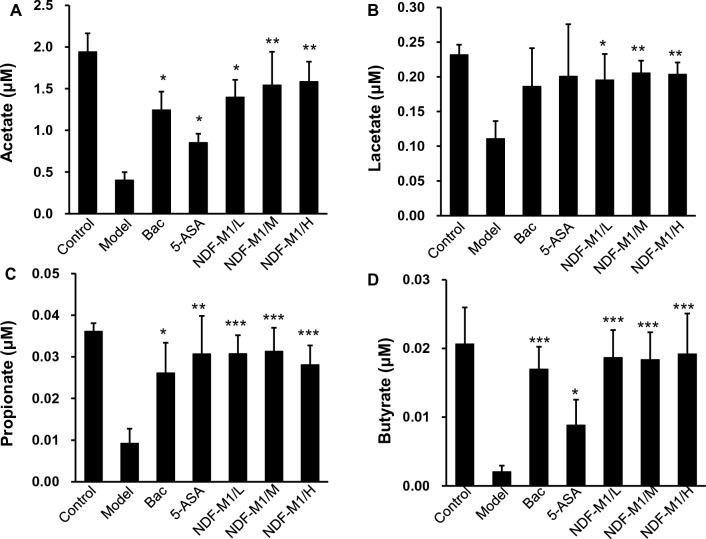
The concentrations of SCFAs. **A**–**D** The concentrations of SCFAs in colon contents. The SCFAs were derivatized and measured by LC − MS/MS. *p < 0.05, **p < 0.01, ***p < 0.001 versus the model group. All data are presented as the mean ± SD (n = 6)

Short-chain fatty acids (SCFAs), produced from the microbial fermentation of indigestible carbohydrates, have been demonstrated to be related to anti-inflammatory effects and a reduced risk of IBD [48]. Thus, we examined the contents of SCFAs in the colon by LC‒MS/MS methods. As shown in Additional file 1: Fig. S5, the results demonstrated that the contents of four SCFAs were significantly increased in the NDF-M1-treated groups compared to the model group and closer to the level of the control group compared to the other groups (Fig. 6). This result was consistent with the Bac fermentation in vitro (Fig. 2G). Taken together, these data suggested that NDF-M1s may exert their anti-inflammatory effects and improve bowel health by shaping the gut microbiota and increasing SCFA production.

To this point, we conducted a preliminary evaluation of the therapeutic effect of NDF-M1s in chronic colitis mice based on body weight, disease index, colorectal length, hematological parameters, biochemical markers, colonic tissue homogenates, inflammatory-related molecules and cells, gut microbiota homeostasis and the contents of SCFAs in colon contents. These results suggested that NDF-M1s played a role in recovering morphological features (body weight, DAI and optical images of the colorectum), maintaining intracellular homeostasis (hematological parameters and biochemical markers), reducing the inflammatory response, maintaining gut microbiota homeostasis and increasing the production of SCFAs. Although some indices did not reach the normal level, they were significantly higher than those in the 5-ASA and Bac groups. Thus, NDF-M1s have significant therapeutic efficacy on chronic colitis in mice.

### The metabolite distribution and targeting performance of NDF-M1/H

**Fig. 7:**
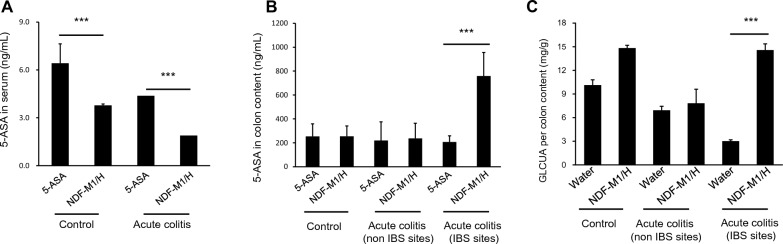
Quantitative analysis of the biodistribution of 5-ASA, proteins and GLCUA in vivo*.* **A** and **B** The production of 5-ASA in serum and colon contents from different sites. Mice with acute colitis induced by 3.0% DSS for 7 days were treated with 5-ASA or NDF-M1/H. Wild-type mice were used as controls and treated identically. Serum was collected to be used for the detection of 5-ASA by LC‒MS/MS. **C** The contents of CLCUA in colon contents from different sites. The IBS site and non-IBS site of mice with acute colitis were collected to detect the levels of 5-ASA, proteins and GLCUA by LC‒MS/MS, BCA Kit and polysaccharide decomposition methods, respectively. ***p < 0.001. All data are presented as the mean ± SD (n = 3)

To further investigate the targeting performance of NDF-M1/H, we firstly evaluated the capability of the NDF-Pro/5-ASA composing NDF-M1/H to capture FITC labeled IL-1β recombinant protein (FITC labeled IL-1β). As shown in Additional file 1: Fig. S6, NDF-Pro/5-ASA with IL-1β antibody exhibited a stronger ability to capture FITC labeled IL-1β than NDF-Pro/5-ASA without IL-1β antibody and thereby validating the targeting performance of NDF-Pro/5-ASA. Subsequently, to explore the action model for NDF-M1/H, the 5-ASA release, changes in protein content and NDF-M1/H metabolite distribution in vivo were systematically studied. Since targeted therapy is largely based on upregulated IL-1β expression in IBS sites, we first verified the level of IL-1β in vivo. As expected, it was threefold higher in the IBS sites than in the non-IBS sites (Additional file 1: Fig. S7A). After the acute colitis mice were treated with NDF-M1/H, 5-ASA, or DI H_2_O for 12 h, we collected the colon contents from the IBS sites and the normal tissue for comparison. Wild-type mice were treated with the same dose of 5-ASA, DI H_2_O or NDF-M1/H as the control groups. As shown in Fig. 7A, the contents of 5-ASA in serum showed a twofold decrease in either wild-type or acute colitis mice with NDF-M1/H treatment compared with those with 5-ASA treatment only. Interestingly, the acute colitis mice with NDF-M1/H treatment had a higher level of 5-ASA in the IBS sites than in the non-IBS sites (Fig. 7B). Moreover, the contents of 5-ASA in the colon contents were detected by a fluorescence quantitative method. Consistent with this, 5-ASA showed dramatic accumulation in IBS sites in mice with acute colitis (Additional file 1: Fig. S7B). Meanwhile, GLCUA was found to be significantly increased in the IBS sites of acute colitis mice after the oral administration of NDF-M1/H compared with other sites (Fig. 7C). Therefore, the engineered NDF-M1/H was capable of delivering 5-ASA at the IBS sites.

**Fig. 8:**
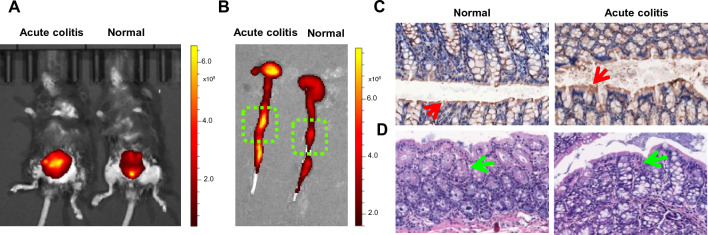
The targeting performance of NDF-M1/H gel microspheres. **A** and **B** In vivo and ex vivo images of NDF-M1/H gel microspheres with Cy5.5 dye in the colorectum. The acute colitis mice model was established by oral administration of 3% DSS for 7 days. Mice with acute colitis were administered an oral gavage of NDF-M1/H gel microspheres with Cy5.5 dye following a 24 h fast. In vivo and ex vivo fluorescence images were obtained by IVIS Spectrum after 24 h treatment (n = 3). **C** Immune staining of IL-1β distributed in the colorectum (red arrows) from the specific region (green boxes). **D** Representative H&E-stained histological sections of colonic tissues from the specific region (green boxes). The degree of inflammatory cell infiltration is delineated (green arrows)

To further study the mechanism of the release, the BSA was replaced with Cy5.5 labeled BSA (Cy5.5-BSA) during the NDF-Pro/5-ASA synthesis. The distribution of NDF-M1/H gel microspheres with Cy5.5 dye in the colorectum could be visualized. As shown in Fig. 8A, the red fluorescence showed robust and aggregation in the colon of acute colitis group compared to the control group after oral gavage of NDF-M1/H gel microspheres with Cy5.5. Subsequently, ex vivo fluorescence imaging of colorectal tissues showed that NDF-M1/H was accumulated at a specific region of colorectum (Fig. 8B). To further verify the inflammatory response in this region, we collected this region to detect the expression level of IL-1β by immunohistochemistry (Fig. 8C) and assess the degree of inflammation using H&E staining (Fig. 8D). As expected, a dramatical inflammatory cell was aggregation and the expression level of IL-1β was increased in the acute colitis group. All above, we studied the four stages of the 5-ASA release in NDF-M1/H delivery system. These results demonstrated the targeting performance of NDF-M1/H.

### The storage stability and chemical stability of NDF-M1/H under different treatments.

To meet expected clinical demands of long-term storage, NDF-M1/H was lyophilized and rehydrated (Additional file 1: Fig. S8A). In addition, lyophilized microsphere powder was also stored at − 80 °C for at least 1 year in this study from past to present. Therefore, we compared the microbial content and the fluorescence curve of NDF-Pro/5-ASA within the lyophilized NDF-M1/H microsphere powder for one year with the freshly prepared NDF-M1/H microspheres. The results in Additional file 1: Fig. S8B and C showed that both microbial activity and fluorescence curve intensity of NDF-Pro/5-ASA demonstrated obviously decrease. To be extremely favorable for clinical translation, we will persistent efforts to improve the storage stability and reduce the difference between before and after freeze-drying by modifying the parameters determining storage stability of lyophilized microspheres.

To further examine the chemical stability of NDF-M1/H under the physiological conditions, we investigated the changes of the microsphere morphology and chemical composition within NDF-M1/H before and after SGIF treatment for 24 h, which did not contain Bac for excluding the interference of Bac fermentation. Firstly, the results of bright-field micrograph in the Additional file 1: Fig. S9A showed that the integrity of the NDF-M1/H microspheres was well maintained for 6 h and then starting to swelling and decomposition. And it was observed that the NDF-Pro/5-ASA was released due to the partial decomposition of the NDF-M1/H microspheres after 24 h. Subsequently, the absorption spectra of NDF-Pro/5-ASA and the protein content were measured. The results in Additional file 1: Fig. S9B and S9C showed that the protein contents were limited reduction and the absorption spectra were similar to that before SGIF treatment. Therefore, we considered that NDF-Pro/5-ASA in NDF-M1/H exposed to SGIF remained stability.

## Discussion

DFs are the polysaccharide portion of food that is not digested in the human small intestine and can be subdivided into water-soluble and water-insoluble fractions [49]. Water-soluble DFs accounted for > 40% of the total DFs in oats and were mainly composed of neutral sugars, accounting for 98%, and β-glucan accounted for at least 80% of the total neutral sugars [50]. Due to its pronounced viscosity, β-glucan derived from oats has physiological activity in the body, such as decreasing the glycemic index of foods and reducing the incidence of cardiovascular disease in people [33, 51]. In this study, dietary fibers were studied as a whole and prepared nanoscale sizes to improve the drug loading efficiency and make them broken down easily by gut microorganisms.

As a kind of natural high molecular carbohydrate compound, oat powder cellulose composed mainly of β-glucan can be obtained by activated enzymatic hydrolysis, and nanoscale oat powder cellulose can be obtained by further oxidation and decomposition of microcrystalline cellulose [52, 53]. Compared to traditional microcrystalline cellulose, nanoscale cellulose has a diameter of 4–20 nm and a length of 0.1–10 μm [54]. Nanoscaled cellulose not only has the physiological properties of cellulose (nondegradation, microbial fermentation properties, high water retention) but also has the properties of nanoparticles (large aspect ratio and mesh structure, high mechanical properties, high expansion surface area, high aspect ratio, better hygroscopicity and rheological property) [55]. Thus, nanoscale cellulose has very high value in high-end composite materials, biomedicine, the food industry and other application fields.

This project is the first to propose a codelivery strategy by designing an anti-IL-1β monoclonal antibody as the targeted molecule and BSA as the linker to covalently bind 5-ASA to the NDF. 5-ASA can be efficiently conjugated with BSA via electrostatic interactions without affecting its activity. Notably, alginate-based microspheres were engineered to encapsulate NDF-Pro/5-ASA and probiotics, which can not only provide strong protection from the adverse acidic and enzymatic environment in the stomach but also deliver 5-ASA to IBS precisely. Although the NDF-M1s exhibited a higher therapeutic effect than the other treatment groups, the results of analysis of variance (ANOVA) showed that the individual differences in the NDF-M1 treatment group were obvious. It was speculated that the delivery system of microspheres might be inhomogeneous during oral administration and gastrointestinal release. Hence, engineering smaller homogeneous microspheres or nanogels as precise delivery systems may achieve a higher and more uniform drug utilization rate.

## Conclusion

In summary, we tailored a precise delivery system by a gel microsphere encapsulating NDF-Pro/5-ASA and Bac to treat mice with chronic colitis. In terms of therapeutic effects, we achieved the aims that NDF-M1 improved the balance of gut microbiota, induced the increase of SCAF productions and effectively alleviated DSS-induced inflammation response in mice. In addition, in the therapeutic mechanism, it provided a protective role in the stomach by gel microspheres, a targeted effect in IBS by IL-1β monoclonal antibodies, and controlled release of 5-ASA in IBS as well as gut microbiota remodeling by fermentation. Therefore, we think it is a promising strategy for the development of site-specific therapy in IBD.

### Supplementary Information

**Additional file 1: Table S1.** Hydrodynamic length and zeta-potential of NDF-Pro/5-ASA. Table S2. The correlation analysis. **Figure S1.** The synthesis and characterizations of NDF-M. **A** and **B** FTIR spectra of NDFs and NDFs-Pro/5-ASA. FTIR spectra were recorded to indicate the existence of surface functional carboxyl groups at about 1590 cm^−1^ and imino groups at about 1560 cm^−1^ using an ATR-FTIR spectrometer at a range of 500 ~ 4500 cm^−1^. **C** and **D** The binding efficiency of IL-1β and BSA on the surface of NDFs. Firstly, 10 μg IL-1β were bound on the surface of NDFs by an amidation using EDC as the coupling agent and NHS as the activator. Secondly, different concentration of BSA was further used to label NDFs by the same method. **E** and **F** The fluorescence spectra at 300–600 nm and the fluorescence intensity at 500 nm of different concentration of 5-ASA in the presence of 1.0 mg/mL BSA. 1.0 mg/ml BSA was separately mixed with different concentration of 5-ASA for 72 h. The mixture was analyzed to observe the change of fluorescence spectra by a Microplate Reader. All data are presented as mean ± SD (n = 3). **Figure S2.** Measurements of short-chain fatty acid (SCFA) concentrations in fermentation broth. **A** The standard curve of SCFAs including propionate, acetate, butyrate, lactate, valerate and isovalerate. **B**–**E** The detection of SCFAs from in vitro fermentation broth of NDF-Ms. NDF-Ms were exposed to SGIF for 24 h and the fermentation broths were collected to detect the concentration of SCFAs, which were derivatized and measured by LC–MS/MS. **F**–**J** The fragmentations of the parent ion of different SCFAs from in vitro fermentation broth of NDF-M1. Red arrows represent the parent ion of different SCFAs detected by LC−MS/MS. All data are presented as mean ± SD (n = 3). **Figure S3.** The toxicity assessment of NDF-Ms both in vitro and in vivo. **A** Cytotoxicity of fermentation broths from NDF-Ms in SW 480 and RAW 264.7 cells. **B** The expression levels of IL-1β and TNF-α in RAW 264.7 cells. NDF-Ms were exposed to SGIF for 24 h and the fermentation broths were collected and incubated with different cells for 24 h to detect the levels of cytokines by ELISA kits and cytotoxicity by MTS kits. Al(OH)_3_ nanoparticle was used as an agonist for the production of IL-1β and TNF-α. **C** H&E staining of liver, spleen and kidney of mice after NDF-M1/H exposure. The control mice were exposed to NDF-M1/H by oral administration or 0.9% saline. After 24 h, the tissue organs including heart, liver, lung, spleen and kidney were collected and fixed in formalin solution for H&E staining. All data are presented as mean ± SD (n = 3). **Figure S4.** The evaluation of hematological parameters and serum biochemical markers. **A**–**D** Hematological parameters were analyzed, including Pltc, Neu, Lym, Mon and Eos blood cells. E–G) Biochemical markers were analyzed, including total proteins, ALT/AST, CRE, UREA, GLU, CHOL, TRIGL, HDL and LDL. C57BL/6 mice (six animals per group) with DSS-induced chronic colitis were treated with NDF-M1s (NDF-M1/L, NDF-M1/M and NDF-M1/H), 5-ASA and Bac by oral administration according to the scheme of treatment procedures as shown in Fig. 3A. Mice were sacrificed to collect blood samples on day 31 for the tests of hematological parameters and serum biochemical markers. All data are presented as mean ± SD (n = 6). **Figure S5.** Measurements of SCFA concentrations from in vivo intestinal lavage fluid. **A** The detection of SCFAs from in vivo intestinal lavage fluid. C57BL/6 mice (six animals per group) with DSS-induced chronic colitis were treated with NDF-M1s (NDF-M1/L, NDF-M1/M and NDF-M1/H), 5-ASA and Bac by oral administration according to the scheme of treatment procedures as shown in Fig. 3A. Mice were sacrificed to collect intestinal lavage fluid for the test of SCFAs, which were derivatized and measured by LC-MS. **B** The fragmentations of the parent ion from different SCFAs. Red arrows represent the parent ion of different SCFAs detected by LC − MS/MS. **Figure S6.** The capability of the NDF-Pro/5-ASA to capture FITC labeled IL-1β. To investigate the ability to capture FITC labeled IL-1β for NDF-Pro/5-ASA, NDF-Pro/5-ASA was coincubated with FITC labeled IL-1β for 24 h in accordance with instruction of ELISA. NDF-Pro/5-ASA without IL-1β antibodies was as the control group. Representative images under bright field and green fluorescent were obtained using a fluorescent microscope (Eclipse Ti-S, Nikon, Japan). **Figure S7.** The expression levels of cytokines and the quantitative analysis of 5-ASA in vivo. **A** The expression levels of cytokines (IL-1β and TNF-α). **B** The quantitative analysis for 5-ASA. The control mice and mice with acute colitis induced by 3.0% DSS for 7 days were treated with 5-ASA or NDF-M1/H, respenctively. IBS site and non IBS site of mice with acute colitis were collected to detect the levels of cytokines (IL-1β and TNF-α) and 5-ASA by ELISA Kits and fluorescence quantitative method, respectively. *p < 0.05, **p < 0.01, ***p < 0.001 and ns (no significance) versus acute colitis mice group. All data are presented as mean ± SD (n = 3). **Figure S8.** The changes of microbial content and the fluorescence curve of NDF-Pro/5-ASA within the lyophilized microspheres. **A** Representative confocal images of NDF-Ms. NDF-M images were obtained by confocal microscopy to visualize the morphology of gel microspheres and the BSA/5-ASA distributions. **B** Representative images of Bac colonies. Colony forming units (CFUs) were determined at 12 h to visualize the bacterial cells. **C** The fluorescence spectra at 300 ~ 600 nm in the presence of 0.5 mg/mL BSA. The fluorescence spectra were obtained by a Microplate Reader (Synergy NEO HTS, Biotek, USA). **Figure S9.** The stability observation of NDF-M1/H under the physiological condition. **A** The changes of the NDF-M1/H microsphere morphology exposed to SGIF treatment for 24 h. The images were taken by an optical microscope (CKX53, OLYMPUS, Japan) equipped with a CCD camera. **B** The changes of the protein content. Proteins were quantified by a BCA Protein Assay kit. **C** The fluorescence spectra at 300–600 nm in the presence of 2.0 mg/mL BSA. The fluorescence spectra were obtained by a Microplate Reader (Synergy NEO HTS, Biotek, USA).

## Data Availability

The data are available from the corresponding author on reasonable request.
