# Supplementary material for: Designing a microbial fermentation-functionalized alginate microsphere for targeted release of 5-ASA using nano dietary fiber carrier for inflammatory bowel disease treatment
Source: J Nanobiotechnology. 2023 Sep 23;21:344. doi: 10.1186/s12951-023-02097-6 (PMC10517557; doi:10.1186/s12951-023-02097-6)
Supplement: Supplementary file 1 — Additional file 1: Table S1. Hydrodynamic length and zeta-potential of NDF-Pro/5-ASA. Table S2. The correlation analysis. Figure S1. The synthesis and characterizations of NDF-M. A and B FTIR spectra of NDFs and NDFs-Pro/5-ASA. FTIR spectra were recorded to indicate the existence of surface functional carboxyl groups at about 1590 cm−1 and imino groups at about 1560 cm−1 using an ATR-FTIR spectrometer at a range of 500 ~ 4500 cm−1. C and D The binding efficiency of IL-1β and BSA on the surface of NDFs. Firstly, 10 μg IL-1β were bound on the surface of NDFs by an amidation using EDC as the coupling agent and NHS as the activator. Secondly, different concentration of BSA was further used to label NDFs by the same method. E and F The fluorescence spectra at 300–600 nm and the fluorescence intensity at 500 nm of different concentration of 5-ASA in the presence of 1.0 mg/mL BSA. 1.0 mg/ml BSA was separately mixed with different concentration of 5-ASA for 72 h. The mixture was analyzed to observe the change of fluorescence spectra by a Microplate Reader. All data are presented as mean ± SD (n = 3). Figure S2. Measurements of short-chain fatty acid (SCFA) concentrations in fermentation broth. A The standard curve of SCFAs including propionate, acetate, butyrate, lactate, valerate and isovalerate. B–E The detection of SCFAs from in vitro fermentation broth of NDF-Ms. NDF-Ms were exposed to SGIF for 24 h and the fermentation broths were collected to detect the concentration of SCFAs, which were derivatized and measured by LC–MS/MS. F–J The fragmentations of the parent ion of different SCFAs from in vitro fermentation broth of NDF-M1. Red arrows represent the parent ion of different SCFAs detected by LC−MS/MS. All data are presented as mean ± SD (n = 3). Figure S3. The toxicity assessment of NDF-Ms both in vitro and in vivo. A Cytotoxicity of fermentation broths from NDF-Ms in SW 480 and RAW 264.7 cells. B The expression levels of IL-1β and TNF-α in RAW 264.7 cells. NDF-Ms we [file 12951_2023_2097_MOESM1_ESM.docx]

Additional file

**Designing a Microbial Fermentation-functionalized Alginate Microsphere for Targeted Release of 5-ASA Using Nano Dietary Fiber Carrier for Inflammatory Bowel Disease Treatment**

Lei Qiu ^a, #^, Renbin Shen ^a, #^, Lei Wei ^a^, Shujuan Xu ^a^, Wei Xia ^c^, Yan Hou ^c^, Jinxin Cui ^a^, Rong Qu ^a^, Jiale Luo ^a^, Jian Cao ^a^, Jie Yang ^b^, Jing Sun^b,*^, Ronglin Ma ^a, *^,Qiang Yu ^a, *^

^a^ Department of Gastroenterology, The Affiliated Suzhou Hospital of Nanjing Medical University, Suzhou Municipal Hospital, Gusu School, Nanjing Medical University, Suzhou 215002, Jiangsu China

^b^ Institute of Medical Biotechnology, Suzhou Vocational Health College, Suzhou 215009, Jiangsu China

^c^ Department of Pathology, The Second Affiliated Hospital of Soochow University, Suzhou 215004, Jiangsu, People’s Republic of China

# Equal contributions

* Corresponding author.

Dr. Jing Sun Email: [jsun@szhct.edu.cn](mailto:jsun@szhct.edu.cn)

Dr. Ronglin Ma Email: [maronglin573@163.com](mailto:maronglin573@163.com)

Dr. Qiang Yu Email: yuqiang0102sz@163.com Tel: +86-512-62362045

**Table S1. Hydrodynamic length and zeta-potential of NDF-Pro/5-ASA**

| **NPs** | **Size (nm)** | **Zeta potential (mV)** |
| --- | --- | --- |
| NDF-1 | 214 ± 40 | -43.3 ± 1.8 |
| NDF-2 | 692 ± 203 | -38.0 ± 8.7 |
| NDF-3 | 1081 ± 65 | -32.4 ± 4.4 |
| NDF-1-Pro/5-ASA | 488 ± 118 | -0.40 ± 0.2 |
| NDF-2-Pro/5-ASA | 1881 ± 590 | -1.30 ± 0.1 |
| NDF-3-Pro/5-ASA | 2690 ± 508 | -9.40 ± 1.1 |

**Table S2. The correlation analysis**

|  | **Correlation coefficients** | | | | |
| --- | --- | --- | --- | --- | --- |
|  | NDF-M0 NDF-M1 NDF-M2 NDF-M3 | | | | |
| 5-ASA release efficiency (%) and Bac growth | | 0.45 | 0.92 | 0.92 | 0.95 |
| DF decomposition efficiency (%) and Bac growth | | / | 0.81 | 0.86 | 0.79 |
| Proteolytic efficiency (%) and Bac growth | | / | 0.86 | 0.84 | 0.86 |

**F**

**E**

**D**

**C**

**B**

**A**

**Figure S1.** **The synthesis and characterizations of NDF-M**

A) and B) FTIR spectra of NDFs and NDFs-Pro/5-ASA. FTIR spectra were recorded to indicate the existence of surface functional carboxyl groups at about 1590 cm^−1^ and imino groups at about 1560 cm^−1^ using an ATR-FTIR spectrometer at a range of 500 ~ 4500 cm^−1^. C) and D) The binding efficiency of IL-1β and BSA on the surface of NDFs. Firstly, 10 μg IL-1β were bound on the surface of NDFs by an amidation using EDC as the coupling agent and NHS as the activator. Secondly, different concentration of BSA was further used to label NDFs by the same method. E) and F) The fluorescence spectra at 300 ~ 600 nm and the fluorescence intensity at 500 nm of different concentration of 5-ASA in the presence of 1.0 mg/mL BSA. 1.0 mg/ml BSA was separately mixed with different concentration of 5-ASA for 72 h. The mixture was analyzed to observe the change of fluorescence spectra by a Microplate Reader. All data are presented as mean ± SD (n = 3).

**D**

**F**

**J**

**I**

**H**

**G**

**E**

**C**

**B**

**A**

**Figure S2. Measurements of short-chain fatty acid (SCFA) concentrations in fermentation broth**

A) The standard curve of SCFAs including propionate, acetate, butyrate, lactate, valerate and isovalerate. B-E) The detection of SCFAs from *in vitro* fermentation broth of NDF-Ms. NDF-Ms were exposed to SGIF for 24 h and the fermentation broths were collected to detect the concentration of SCFAs, which were derivatized and measured by LC-MS/MS. F-J) The fragmentations of the parent ion of different SCFAs from in vitro fermentation broth of NDF-M1. Red arrows represent the parent ion of different SCFAs detected by LC−MS/MS. All data are presented as mean ± SD (n = 3).

**C**

**A**

**B**

**Figure S3.** **The toxicity assessment of NDF-Ms both *in vitro* and *in vivo*.**

A) Cytotoxicity of fermentation broths from NDF-Ms in SW 480 and RAW 264.7 cells. B) The expression levels of IL-1β and TNF-α in RAW 264.7 cells. NDF-Ms were exposed to SGIF for 24 h and the fermentation broths were collected and incubated with different cells for 24 h to detect the levels of cytokines by ELISA kits and cytotoxicity by MTS kits. Al(OH)_3_ nanoparticle was used as an agonist for the production of IL-1β and TNF-α. C) H&E staining of liver, spleen and kidney of mice after NDF-M1/H exposure. The control mice were exposed to NDF-M1/H by oral administration or 0.9% saline. After 24 h, the tissue organs including heart, liver, lung, spleen and kidney were collected and fixed in formalin solution for H&E staining. All data are presented as mean ± SD (n = 3).

**C**

**G**

**F**

**E**

**D**

**B**

**A**

**Figure S4. The evaluation of hematological parameters and serum biochemical markers**

A-D) Hematological parameters were analyzed, including Pltc, Neu, Lym, Mon and Eos blood cells. E-G) Biochemical markers were analyzed, including total proteins, ALT/AST, CRE, UREA, GLU, CHOL, TRIGL, HDL and LDL. C57BL/6 mice (six animals per group) with DSS-induced chronic colitis were treated with NDF-M1s (NDF-M1/L, NDF-M1/M and NDF-M1/H), 5-ASA and Bac by oral administration according to the scheme of treatment procedures as shown in Figure 3A. Mice were sacrificed to collect blood samples on day 31 for the tests of hematological parameters and serum biochemical markers. All data are presented as mean ± SD (n = 6).

**A**

**B**

**Figure S5. Measurements of SCFA concentrations from *in vivo* intestinal lavage fluid**

A) The detection of SCFAs from *in vivo* intestinal lavage fluid. C57BL/6 mice (six animals per group) with DSS-induced chronic colitis were treated with NDF-M1s (NDF-M1/L, NDF-M1/M and NDF-M1/H), 5-ASA and Bac by oral administration according to the scheme of treatment procedures as shown in Figure 3A. Mice were sacrificed to collect intestinal lavage fluid for the test of SCFAs, which were derivatized and measured by LC-MS. B) The fragmentations of the parent ion from different SCFAs. Red arrows represent the parent ion of different SCFAs detected by LC−MS/MS.

**Figure S6. The capability of the NDF-Pro/5-ASA to capture FITC labeled IL-1β**

To investigate the ability to capture FITC labeled IL-1β for NDF-Pro/5-ASA, NDF-Pro/5-ASA was coincubated with FITC labeled IL-1β for 24 h in accordance with instruction of ELISA. NDF-Pro/5-ASA without IL-1β antibodies was as the control group. Representative images under bright field and green fluorescent were obtained using a fluorescent microscope (Eclipse Ti-S, Nikon, Japan).

**B**

**A**

**Figure S7. The expression levels of cytokines and the quantitative analysis of 5-ASA *in vivo***

A) The expression levels of cytokines (IL-1β and TNF-α). B) The quantitative analysis for 5-ASA. The control mice and mice with acute colitis induced by 3.0% DSS for 7 days were treated with 5-ASA or NDF-M1/H, respenctively. IBS site and non IBS site of mice with acute colitis were collected to detect the levels of cytokines (IL-1β and TNF-α) and 5-ASA by ELISA Kits and fluorescence quantitative method, respectively. *p < 0.05, **p < 0.01, ***p < 0.001 and ns (no significance) versus acute colitis mice group. All data are presented as mean ± SD (n = 3).

**C**

**B**

**A**

**Figure S8. The changes of microbial content and the fluorescence curve of NDF-Pro/5-ASA within the lyophilized microspheres**

A) Representative confocal images of NDF-Ms. NDF-M images were obtained by confocal microscopy to visualize the morphology of gel microspheres and the BSA/5-ASA distributions. B) Representative images of Bac colonies. Colony forming units (CFUs) were determined at 12 h to visualize the bacterial cells. C) The fluorescence spectra at 300 ~ 600 nm in the presence of 0.5 mg/mL BSA. The fluorescence spectra were obtained by a Microplate Reader (Synergy NEO HTS, Biotek, USA).

**A**

**C**

**B**

**Figure S9. The stability observation of NDF-M1/H under the physiological condition**

A) The changes of the NDF-M1/H microsphere morphology exposed to SGIF treatment for 24 h. The images were taken by an optical microscope (CKX53, OLYMPUS, Japan) equipped with a CCD camera. B) The changes of the protein content. Proteins were quantified by a BCA Protein Assay kit. C) The fluorescence spectra at 300 ~ 600 nm in the presence of 2.0 mg/mL BSA. The fluorescence spectra were obtained by a Microplate Reader (Synergy NEO HTS, Biotek, USA).
